# The Hox protein Antennapedia orchestrates *Drosophila* adult flight muscle development

**DOI:** 10.1126/sciadv.adr2261

**Published:** 2024-11-27

**Authors:** Gabriela Poliacikova, Aïcha Aouane, Nathalie Caruso, Nicolas Brouilly, Corinne Maurel-Zaffran, Yacine Graba, Andrew J. Saurin

**Affiliations:** Aix-Marseille Univ, CNRS, Developmental Biology Institute of Marseille (IBDM), UMR 7288, Case 907, Parc Scientifique de Luminy, Marseille Cedex 09 13288, France.

## Abstract

Muscle development and diversity require a large number of spatially and temporally regulated events controlled by transcription factors (TFs). *Drosophila* has long stood as a model to study myogenesis due to the highly conserved key TFs involved at all stages of muscle development. While many studies focused on the diversification of *Drosophila* larval musculature, how distinct adult muscle types are generated is much less characterized. Here, we identify an essential regulator of *Drosophila* thoracic flight muscle development, the Hox TF Antennapedia (Antp). Correcting a long-standing belief that flight muscle development occurs without the input of Hox TFs, we show that Antp intervenes at several stages of flight muscle development, from the establishment of the progenitor pool in the embryo to myoblast differentiation in the early pupa. Furthermore, the precisely regulated clearance of Hox in the developing flight muscle fibers is required to allow for fibrillar muscle fate diversification, setting these muscles apart from all other adult tubular muscle types.

## Teaser

The timely presence and absence of the Hox protein Antp are required for the correct adult flight muscle development.

## INTRODUCTION

Muscle development requires a network of transcription factors (TFs) that diversify distinct muscle types. Studies in *Drosophila* larval somatic muscles have provided an extensive picture of the gene regulatory networks responsible for the muscle progenitor’s specification. The current picture is that, following muscle progenitor specification, dependent on Notch and Ras/MAPK (mitogen-activated protein kinase) signaling pathways, muscle diversity is achieved through a combinatorial expression of identity TFs (iTFs) and *Hox* genes, controlling the muscle identity, such as size, position, innervation, and attachment [reviewed in (*1*)]. In the case of embryonic/larval muscles, *Hox* genes have been shown to intervene at several steps of their development, providing specific identity to muscle precursors by the regulation of iTFs (*2*–*5*), regulating the final progenitor number for a given muscle and later, during development, regulating the final muscle size [(*6*); reviewed in (*7*)].

In contrast to larval muscle precursors, the specification of adult muscle precursors (AMPs) is much less characterized [reviewed in (*8*)]. In this context, it has been shown that mesodermal AbdA overexpression converts thoracic AMPs into abdominal AMPs (*9*), suggesting that *Hox* genes provide identity to both embryonic precursors and AMPs. One particular adult muscle, the *Drosophila* flight muscle, was suggested to develop without any Hox input as precursors of this muscle associated with the larval wing disc do not express any *Hox* gene (*10*). However, both older and recent staining methods of the embryonic thoracic mesoderm and larval wing discs indicate that Antp, the *Hox* gene responsible for mesothoracic identity establishment (*11*), is expressed in territories that may correspond to flight muscle AMPs (*5*, *12*–*14*), leaving the role of whether and how Hox regulate adult flight muscle contentious.

## RESULTS

### Antp is expressed in flight muscle myoblasts

**Fig. 1.**
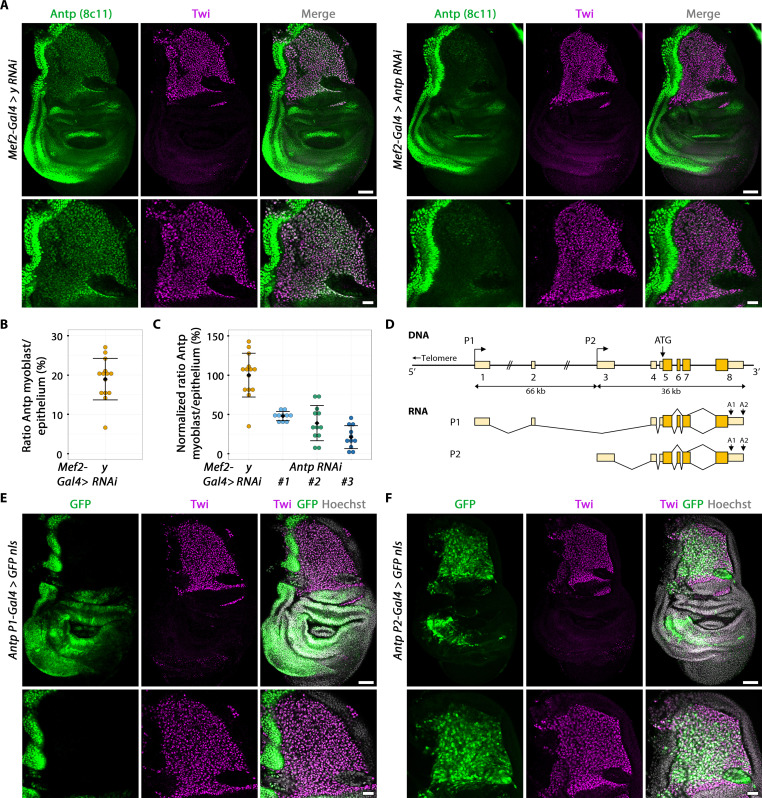
Antp is expressed in flight muscle myoblasts from the P2. (**A**) Confocal sections of 0-hour APF wing discs expressing *UAS-y RNAi* (left) or *UAS*-*Antp RNAi* #1 (right) driven by *Mef2-Gal4*, stained with anti-Antp (8c11) and anti-Twi. (**B**) Ratio of Antp intensity in the myoblast area versus anterior epithelium. The mean and SD are shown. Each dot represents a value from one disc (*n* = 14 discs from at least seven animals). (**C**) Normalized Antp intensity under *Antp RNAi* conditions (each RNAi line is depicted by with a “#”). Each dot represents a value from one disc (*n*_WT_ = 14, *n*_#1_ = 10, *n*_#2_ = 12, and *n*_#3_ = 11 wing discs from at least five animals). (**D**) *Antp* gene and transcripts (coding exons are dark yellow, and noncoding exons are light yellow smaller boxes). P1 and P2 represent two distinct promoters, and A1 and A2 represent two alternative polyadenylation sites. (**E** and **F**) Confocal sections of 0-hour APF wing discs expressing *UAS-GFP nls* driven by *Antp P1-Gal4* (E) or *Antp P2-Gal4* (F), stained with anti-GFP and anti-Twi. Scale bars, 50 μm (top view) and 20 μm (bottom view).

At the end of embryogenesis, flight muscle precursors (myoblasts) attach to the larval wing imaginal discs, precursors of the adult wings (*15*). To unambiguously demonstrate that Antp is expressed in flight muscle myoblasts, we labeled larval wing discs with antibodies against Twist (Twi) (a marker of myoblasts) and Antp at the onset of pupation [0 hours after puparium formation (APF)], where the number of myoblasts is the highest (*16*). We detected strong Antp expression in the anterior disc epithelium (Fig. 1A) (*14*, *17*), and we also observed weaker expression, ~75% lower compared to the disc epithelium (Fig. 1B), in the notum region colocalizing with Twi expression, showing that Antp is expressed in flight muscle myoblasts. To demonstrate specificity of this staining, we down-regulated Antp expression in myoblasts using three different *UAS-Antp* RNA interference (RNAi) lines, driven by a muscle-specific *Mef2-*Gal4 driver, active in wing disc–associated myoblasts from the third instar larval stage (*18*) and observed reduction in Antp expression (Fig. 1, A and C). We additionally confirmed Antp expression in myoblasts with a second distinct antibody against Antp, with a green fluorescent protein (GFP) reporter driven by an *Antp-*Gal4 line, and a GFP insertion into the *Antp* locus, fusing GFP to Antp (fig. S1, A to C).

The locus of the *Antp* gene is large, spanning over 100 kb of genomic distance and contains two internal promoters, promoter 1 (P1) and promoter 2 (P2), that drive *Antp* expression in spatially different locations (schematized in Fig. 1D) (*19*–*21*). To determine which promoter drives Antp expression in flight muscle myoblasts, we used a *P1-*Gal4 line and constructed a *P2-*Gal4 line using minimal regulatory elements of the P2 promoter described in (*22*). In agreement with previous reports (*13*, *14*), we found that the P1 promoter drives the reporter expression in the anterior disc epithelium and the wing pouch (Fig. 1E). The P2 promoter, on the other hand, shows strong expression in most myoblasts and no expression in the disc ectoderm, being thus in the wing disc, a mesoderm-specific promoter (Fig. 1F). Together, these data demonstrate that Antp is expressed in wing disc–associated myoblasts, precursors of adult flight muscles, and this expression is driven by the P2 promoter.

### Antp loss leads to the absence of adult flight muscle precursors

Having established that Antp is expressed in wing disc–associated myoblasts, we asked what role Antp plays during adult flight myogenesis. *Antp* homozygous null mutations are lethal during embryogenesis resulting from homeotic transformation of the mesothorax (second thoracic segment T2) toward a prothorax identity (first thoracic segment T1) (*23*). However, this transformation and lethality can be rescued by providing a single dose of Antp from the P1 promoter: When a P2-specific mutant line *Antp^s1^* is heterozygous with a null mutation of *Antp* (*Antp^ns-rvc3^*), embryos and larvae develop normally, giving viable animals until the late pupal stage (*24*). We thus used this genetic combination, which we refer to as *Antp^P2-null^* for simplicity, to study Antp function driven from the P2 promoter and its role in flight myogenesis.

**Fig. 2.**
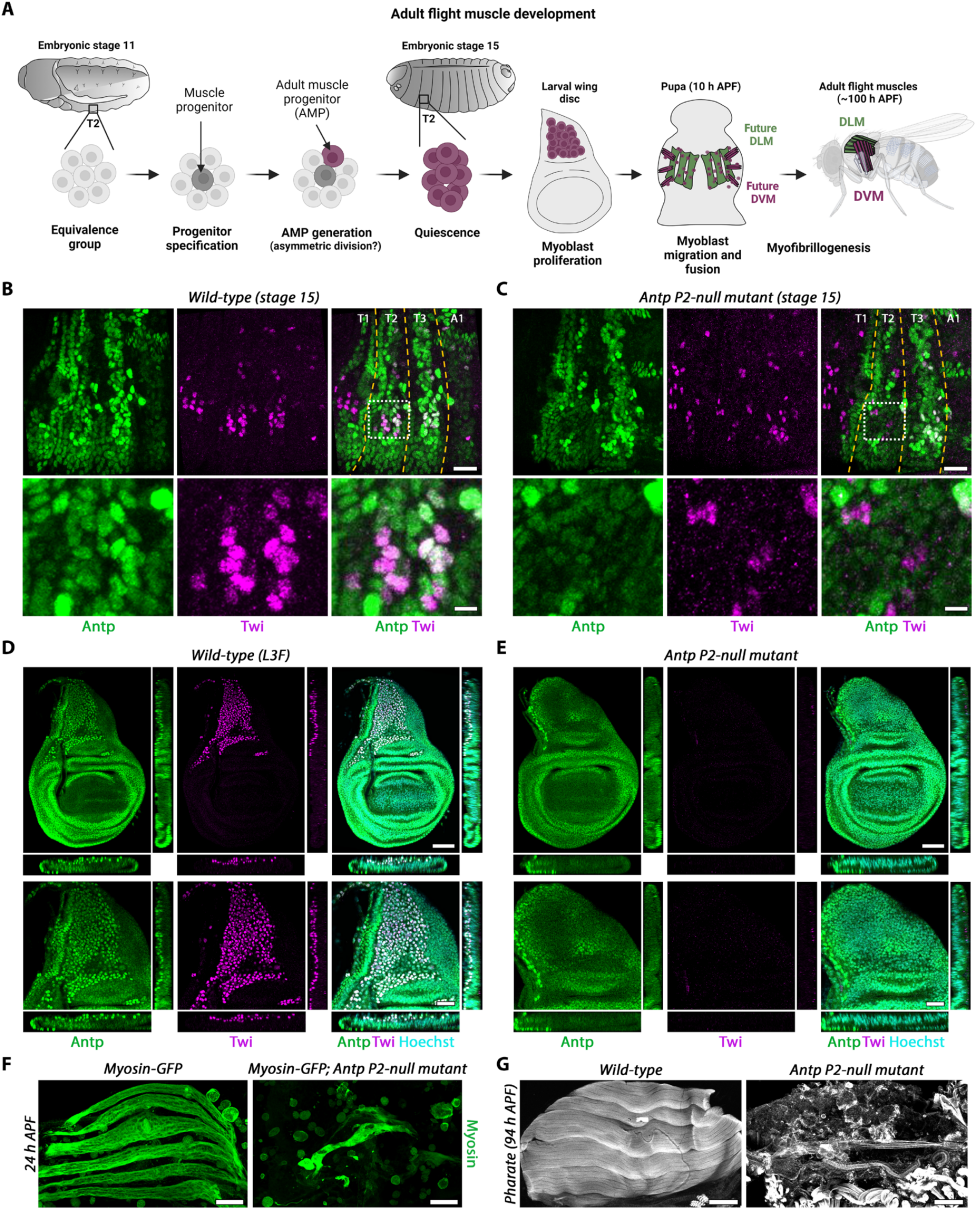
*Antp* mutation leads to the absence of adult flight muscles. (**A**) Schematic representation of adult flight myogenesis. (**B** and **C**) Confocal sections of stage 15 embryos labeled with antibodies against Antp and Twi. Genotypes are (B) WT and (C) *Antp^P2-null^* mutant: *Antp^nsrv-c3^* (null mutation) over *Antp^s1^* (P2 mutation). T1, T2, and T3 depict the three thoracic segment, and A1 depicts the first abdominal segment. Scale bars, 20 μm (large views) and 5 μm (zoomed views on T2 AMPs). (**D** and **E**) Confocal sections of wing discs at the L3 feeding stage labeled with antibodies against Antp and Twi. Genotypes are identical to (B) and (C). Scale bars, 50 μm (large views) and 25 μm (zoomed views). (**F**) Confocal projections of 24-hour APF pupal IFMs labeled with GFP to visualize myosin. Genotypes are identical to (B) and (C) with *weeP26* transgene (Myosin-GFP) added. Scale bars, 50 μm. (**G**) Confocal sections of pupal IFM at the pharate stage, labeled with phalloidin. Genotypes are identical to (B) and (C). Scale bars, 100 μm. DLM, dorsal longitudinal muscles; DVM, dorsal-ventral muscles; h, hours.

*Drosophila* somatic myogenesis is a two-stage process where larval muscles are generated during embryogenesis and AMPs, specified in the embryo from a common lineage of cells to larval muscle, proliferate in the larvae, and differentiate into adult muscles during pupal stages (Fig. 2A). AMPs in the embryo are distinguished from larval muscle precursors by persistent Twi expression from stage 14 onward (*15*). Flight AMPs develop in T2, where they are mixed with leg muscle precursors and are visible as a patch of Twi-positive cells at the ventral side of the embryo at stage 15 onward. At this stage, Antp expression can be found in T2 and T3 AMPs, with a lower expression in the T2 segment (Fig. 2B). Notably, in the *Antp^P2-null^* mutant, embryos showed a drastically reduced number of T2 AMPs (Fig. 2C), while heterozygous flies for any of the *Antp* mutant alleles showed no visible phenotype (fig. S2A). AMPs in the T3 segment were not affected in the *Antp^P2-null^* mutant, suggesting that the expression of Antp in the T3 AMPs is driven by the P1 promoter, which has been previously reported (*12*, *25*) and confirmed by our staining (fig. S2B). These results suggest that, in the absence of *Antp* expression from the P2 promoter in the embryonic T2 mesoderm, flight muscle AMPs are not specified or are eliminated shortly after their specification.

Flight muscles are composed of indirect flight muscles (IFMs) that power wing stroke and direct flight muscles (DFMs) that regulate the wing angle. The IFM, which we will for simplicity refer to as “flight muscles,” are themselves composed of dorsal longitudinal muscles (DLMs), which are responsible for wing depression, and dorsal-ventral muscles (DVMs), which are responsible for wing elevation. Both DFMs and IFMs develop from wing disc–associated myoblasts (*26*), which themselves derive from T2 embryonic AMPs (*15*). We thus looked at myoblasts at the L3 larval stage because, if AMPs in the embryo are truly absent in the *Antp^P2-null^* mutant, then the wing discs should be devoid of myoblasts at the larval stage.

While heterozygous larvae for any of the two *Antp* mutant alleles showed no visible phenotype (fig. S2C), in the *Antp^P2-null^* homozygous mutant wing discs, the whole myoblast population, which would normally give rise to IFMs and DFMs, was absent, as shown by a lack of Twi staining and a physical absence of the myoblast layer on the top of the notum epithelium (Fig. 2, D and E). To permit adult muscle development, most larval muscles histolyze at the onset of pupation. The larval oblique muscles (LOMs) 1, 2, and 3, however, persist and serve as scaffolds for DLM development, whereby wing disc myoblasts fuse with them in the early stages of pupation (*27*). However, these templates degenerate if there are no fusing myoblasts (*28*) in the early pupa, which we also observe in the *Antp^P2-null^* mutant (Fig. 2F). This results in pharate animals whose thorax is completely devoid of any flight muscle (Fig. 2G). Heterozygous animals develop flight muscles normally (fig. S2, D and E). Overall, these results show that the P2-specific loss of *Antp* leads to a complete absence of adult flight muscles due to lack of flight muscle AMPs and thus places *Antp* as a key player in early adult flight myogenesis.

### Antp regulates flight muscle splitting and myoblast fusion

**Fig. 3.**
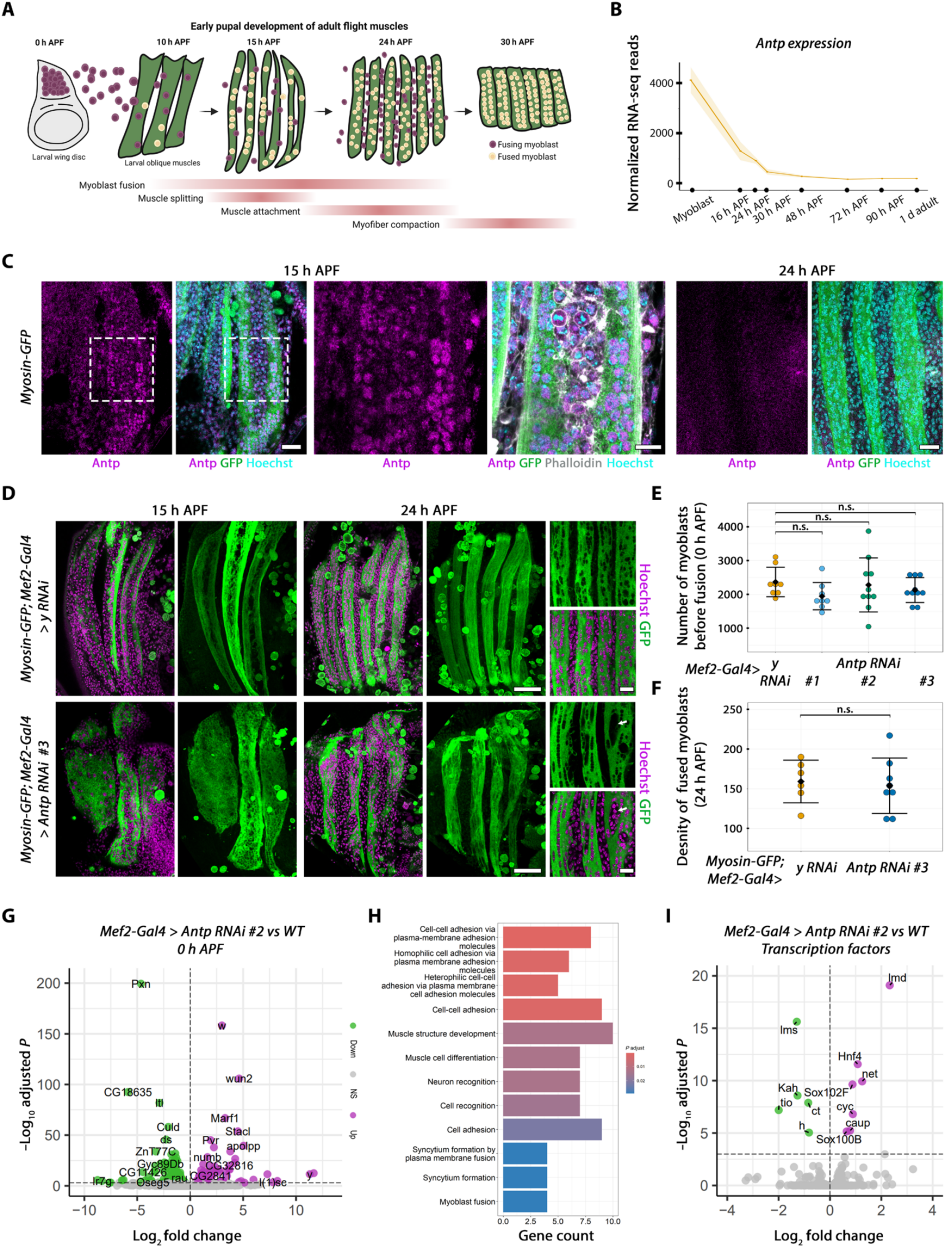
*Antp* regulates flight muscle differentiation. (**A**) Schematic representation of pupal flight myogenesis. d, day. (**B**) *Antp* mRNA normalized expression, analyzed from (*29*). (**C**) Confocal sections of 15- and 24-hour APF flight muscles expressing *Myosin-GFP*, stained with anti-Antp, anti-GFP (to visualize myosin), phalloidin, and Hoechst. Scale bars, 20 μm [large views (left)] and 10 μm [zoomed views (right)], depicted by the white dashed box. (**D**) Confocal sections (left) and projections (right) of 15- and 24-hour APF flight muscles expressing *Myosin-GFP*; *Mef2-Gal4* driving *UAS-y RNAi* as control (top) or *UAS-Antp RNAi #3* (bottom), stained with anti-GFP and Hoechst. Scale bars, 50 μm (large sections) and 15 μm (zoomed views). Arrow depicts zones of high nuclei density. (**E**) Quantification of the number of myoblasts in 0-hour APF wing disc upon *Antp* KD. Mean and SD are shown; each dot represents a value from one disc (*n*_WT_ = 8, *n*_#1_ = 8, *n*_#2_ = 10, and *n*_#3_ = 10 wing discs from at least five animals). n.s., not significant. (**F**) Same as in (E) at 24 hours APF (*n*_WT_ = 6 and *n*_#3_ = 8 animals). (**G**) Volcano plot of DEGs between WT and *UAS-Antp RNAi #2* driven by the *Mef2-Gal4* driver. n.s., not significant. (**H**) GO analysis using significantly up-regulated genes. (**I**) Volcano plot of TFs, the same conditions as (G).

Following myoblast fusion with the larval templates in the early stages of pupation, the three LOM templates split to give rise to the final DLM pattern of six fibers (see Fig. 3A) (*27*). A transcriptional timecourse study of flight myogenesis detected *Antp* transcripts at the myoblast stage and up to 24 hours APF when myoblast fusion is almost completed (Fig. 3B) (*29*). We confirmed these results at the protein level with antibody staining, showing that, at 15 hours APF, Antp is expressed in myoblasts undergoing fusion, located outside the fiber, making contacts with the myotube membrane, and inside, at the fiber periphery (Fig. 3C). Antp expression is weaker in myoblasts located inside the myotube and can no longer be detected at 24 hours APF, meaning that Antp is down-regulated in myoblasts a few hours after their fusion.

In addition to its role in flight muscle AMP specification, we thus asked whether *Antp* has a role in these early stages of muscle differentiation. Because the *Antp^P2-null^* mutant condition results in the absence of myoblasts at the larval stage, we used a UAS/Gal4 system to down-regulate *Antp* expression during myogenesis using the *Mef2-*Gal4 driver (see Fig. 1D). Mef2 expression is absent in AMPs in the embryo (*30*), with expression turning on in myoblasts at larval third instar stages, allowing for *Antp* knockdown (KD) in proliferating myoblasts, without affecting their specification in the embryo. We observed that, upon *Mef2*-Gal4–driven *Antp* KD, LOM splitting is markedly delayed and often incomplete with fewer muscle fibers at the end of the splitting process compared to control (Fig. 3D) accompanied by a decrease in the muscle compaction (fig. S3, A and B). To gain more insight into the dynamics of this process, we set up a live imaging of the muscle splitting using a *UAS-Gma::GFP* transgene (*31*) labeling actin, driven by the *Mef2-*Gal4 driver (movie S1) (*32*). We observed that, under this *Antp* KD condition, the delay in the splitting is accompanied by a delay in myoblast fusion, where myoblasts appear to form the initial actin-rich patch but fail to complete the fusion for several hours (fig. S3C, ex. 1, and movie S2). While the number of myoblasts prior (Fig. 3E) or after fusion (Fig. 3F) remains unchanged, we observed that nuclei were often improperly interspaced and often contained large hollow pockets (Fig. 3D, arrow). Furthermore, in an apparently stochastic manner, some muscle fibers became detached and were eliminated before the compaction stage, likely accounting for the decreased number of muscle fibers we observed in immunofluorescence experiments (fig. S3C, ex. 2, and movie S3).

At the adult stage, *Mef2*-Gal4–driven *Antp* KD results in numerous flight muscle phenotypes: (i) a held-out wing phenotype (fig. S4A) that does not support flight (fig. S4B); (ii) a decrease in adult DLM fiber number, which nevertheless fill the whole thorax; and (iii) phenotypes in sarcomeric compaction, fiber splitting, and Z-line alignment (fig. S4C), with sarcomeres being significantly shorter and thicker (fig. S4D). The strongest RNAi line #3 led to a pharate lethality, displaying the same abovementioned phenotypes (fig. S4, E to G). An incorrect adult fiber patterning as well as defaults in sarcomeric integrity were also observed in the DVMs (fig. S4H). We confirmed these phenotypes with the *Him*-GAL4 line, active only until myoblast fusion (*29*), which, in this case, behaves similarly to the *Mef2*-GAL4 line, in combination with *Antp* RNAi #2, leading to patterning defects (fig. S5A). We also tested another flight muscle–specific GAL4 line, *1151-*Gal4, which, similarly to *Mef2-*Gal4, is active in larval myoblasts until the adult stage (*33*). This line in our hands has weaker expression, with only the strongest *Antp* RNAi #3 leading to patterning phenotypes (fig. S5B). As expected from Antp expression during early pupal stages, driving *Antp* KD after fusion (starting at ~30 hours APF) with *Act88F-*Gal4 (*29*) does not perturb adult flight myogenesis (fig. S5C). These phenotypes generated by *Antp* RNAi are specific because they were rescued by simultaneously overexpressing Antp (fig. S6). Together, these results show that Antp expression in early pupal myoblasts is required for their correct fusion, muscle splitting, and correct early sarcomerogenesis.

To gain molecular insights underlying the role of *Antp* in flight myogenesis, we performed an RNA sequencing (RNA-seq) analysis on fluorescence-activated cell sorting (FACS)–sorted 0-hour APF myoblasts (data S1). Using a highly stringent significance threshold (adjusted *P* < 0.001), we found 236 differentially expressed genes (DEGs) between *Mef2*-Gal4–driven *Antp* KD and the control condition, 56% of which were up-regulated and 44% down-regulated (Fig. 3G). Gene ontology (GO) analysis showed that up-regulated genes, i.e., genes repressed by Antp in myoblasts, associate with cell-cell adhesion, muscle differentiation, and fusion processes (Fig. 3H and data S1). This suggests that *Antp* KD may lead to premature differentiation, likely accounting for the deficit in myoblast fusion and splitting we observed, which is similarly observed through perturbing the levels of many key genes responsible for muscle differentiation, such as *Notch*, *twi*, and *Mef2* (*33*–*35*). Down-regulated genes upon *Antp* KD, i.e., genes activated by Antp in myoblasts, were associated with not only BMP signaling but also cell-cell adhesion (fig. S7A and data S1). Among the 236 DEGs, 15 encode for TFs (Fig. 3I and data S1), all linked with cell differentiation and/or specification, including TFs known to act in muscle development, such as *lame duck* (*lmd*) (*36*), *lateral muscle scarcer* (*lms*) (*37*), *optomotor blind-related-gene 1* (*org-1*) (*38*), *cut* (*ct*) (*26*), and *Kahuli* (*Kah*) (*39*). Antp thus controls a potential large gene regulatory network in adult flight muscle myoblasts.

To further endow functionality to our RNA-seq dataset, we correlated them with data obtained from a functional flight test RNAi screen to identify factors involved in adult flight muscle development (*40*). This led to the identification of 17 genes down-regulated upon *Antp* KD that have a phenotype in the flight screen (fig. S7B and data S2). Toward a potential function in sarcomerogenesis, one gene whose down-regulation caught our attention is the *Mlc2* gene as its mutation was shown to cause flight muscle sarcomeric phenotypes highly similar to *Antp* KD (*41*) and recessive mutations of its vertebrate homolog, MYL2, were associated with infantile muscle fiber disease and cardiomyopathy, displaying similar sarcomeric phenotypes [(*42*); reviewed in (*43*)]. Certain sarcomeric phenotypes, notably the discontinuous Z-disk observed upon *Antp* KD, can be rescued by *Mlc2* overexpression (fig. S7C).

Together, the study of Antp function in early pupal stages shows that, besides its role in the establishment of flight muscle AMPs in the embryo (Fig. 2), Antp also plays a role at the early pupal stage, promoting myoblast transcriptional changes and controlling myoblast fusion, muscle splitting, and correct early sarcomerogenesis.

### Antp developmental clearance is necessary for fibrillar fiber type specification

**Fig. 4.**
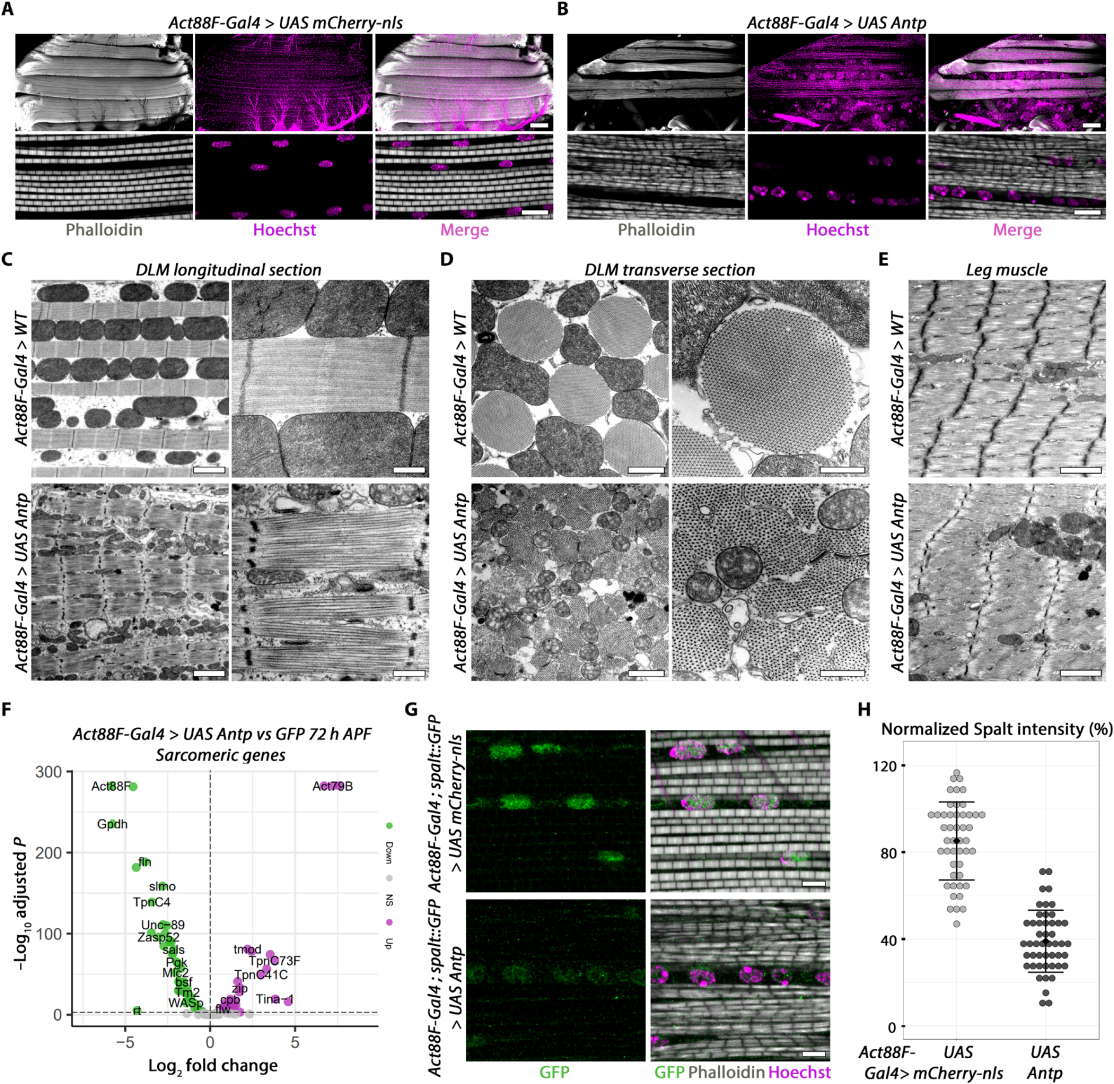
*Antp* overexpression suppresses fibrillary muscle fate. (**A** and **B**) Confocal sections of adult female DLMs stained with phalloidin and Hoechst (top), with zoomed views on sarcomeres (bottom). Scale bars, 100 μm (large views) and 10 μm (zoomed views). Genotypes are *UAS-mCherry-nls* (A) and *UAS-Antp* (B) driven by the *Act88F-Gal4* driver. (**C**) Transmission electron microscopy (TEM) micrographs of adult DLMs, cut in longitudinal sections. Scale bars, 2 μm (large views) and 500 nm (zoomed views). (**D**) Same as (C), cut in cross sections. Scale bars, 1 μm (large views) and 500 nm (zoomed views). (**E**) TEM micrographs of leg muscles. Scale bars, 2 μm. For (C) to (E), genotypes are WT (top) and *UAS-Antp* (bottom) driven by the *Act88F-Gal4* driver. (**F**) Volcano plot of sarcomeric DEGs between *UAS-GFP* and *UAS-Antp* driven by the *Act88F-Gal4* driver. (**G**) Confocal sections of adult female DLMs stained with anti-GFP (to visualize Salm), phalloidin, and Hoechst. Scale bars, 5 μm. (**H**) Quantification of normalized Salm intensity; represented is the mean and SD where each point represents a value of a single nucleus (*n*_mcherry-nls_ = 60 nuclei and *n*_Antp_ = 48 nuclei from at least three animals). Genotypes in (G) and (H) are identical to (A) and (B), with the *spalt::GFP* transgene added.

Antp expression sharply decreases following myoblast fusion (Fig. 3, B and C). To address whether Antp clearance is functionally important, we maintained its expression after fusion with a late, flight muscle–specific *Act88F-*Gal4 driver, active from around 24 hours APF (*29*). Notably, using light and electronic microscopy, we observed that Antp maintenance leads to sarcomeric perturbations (Fig. 4, A to D). Adult *Drosophila* has two types of muscle fiber: stretch-activated fibrillar muscle composed of individual myofibrils and calcium-activated tubular muscle with laterally aligned myofibrils [reviewed in (*8*)], with IFMs being the only fibrillar muscle type. In maintaining Antp expression, the IFM myofibrils seem to adopt a tubular-like fate, resembling morphologically leg muscles, with myofibrils aligning along the Z-disk and lesser mitochondrial volume and distribution (compare Fig. 4E with Fig. 4C, bottom).

To molecularly study whether Antp maintenance alters muscle fiber fate, we performed RNA-seq analysis at 72 hours APF, the earliest stage with a visible sarcomere perturbation (fig. S8, A and B, and data S3). Maintaining Antp expression during late myogenesis led to a very large number of DEGs (4264 genes; adjusted *P* < 0.001), from which 109 were related to the sarcomere structure (Fig. 4F and data S3) (*44*). Among the most significantly down-regulated sarcomeric genes, we found several fibrillar-specific genes, such as *Act88F*, whereas among the most significantly up-regulated sarcomeric genes, we found several tubular-specific genes, such as *Act79B* (fig. S8C) (*45*, *46*). These data demonstrate that Antp maintenance prevents acquisition of the fibrillar fiber type. In favor of this is that, among the repressed genes upon maintained *Antp* expression, we found *spalt major* (*salm*), a master specifier of fibrillar muscle type (*47*), already reported as a negative target of Antp in the antenna disc (fig. S8D) (*48*), which we show also holds true by immunofluorescence in the flight muscle (Fig. 4, G and H). The activation of *salm* expression in postfusion myoblasts was shown to be dependent on the transcriptional activator *vestigial* (*vg*) (*47*). We thus conducted an epistatic experiment between *vg* and *Antp*, showing that, while *vg* overexpression alone does not seem to perturb IFM development (*26*), its simultaneous overexpression with *Antp* does not rescue *salm* repression nor rescue the tubular-like IFM muscle fate caused by *Antp* gain-of-function (fig. S8E). We thus conclude that *salm* repression by *Antp* is independent of *vg*. Salm expression starts in myotubes only after myoblast fusion is completed (*29*, *47*), at the time that coincides with Antp down-regulation (see Fig. 3B). It is thus tempting to speculate that that *Antp* acts as direct or indirect repressor of *salm* expression, resulting in the tubular fiber muscle type in adult muscles whenever Antp expression is maintained. DFMs are of tubular muscle type and originate from myoblasts on the ventral side of the wing disc notum (*26*), which we showed express Antp at the larval stage (Fig. 1). Unlike in the IFMs, we detect persistent Antp expression in adult DFMs (fig. S8F), reinforcing our hypothesis that maintained Antp expression favors tubular muscle fate through lack of *salm* expression.

It has been reported that, in the haltere disc, the Hox protein Ultrabithorax (Ubx) binds to the *salm* promoter (*49*), where it negatively regulates a *salm* cis-regulatory element (*50*) and that Ubx overexpression in the thoracic mesoderm represses *Act88F* (*51*). We thus probed whether the expression of *Hox* genes, in general, also leads to similar muscle type transformation as observed using Antp. Notably, the expression of any *Hox* gene in the developing IFM led to various degrees of flight muscle transformation toward tubular-like fate as well as more or less strong *salm* repression (fig. S9), suggesting that fibrillar fiber type suppression is shared by all Hox paralogs and can thus be considered as a Hox generic function [reviewed in (*52*)].

## DISCUSSION

The specification of different muscle fiber fates in *Drosophila* involves complex networks of TFs, among which iTFs and *Hox* genes. Challenging previously published results (*10*), in this study, we show that the specification of thoracic flight musculature is dependent on the *Hox* gene *Antp*. As suggested previously (*5*), we believe that the discrepancy in *Antp* expression in thoracic myoblasts results from the difficulty of detecting low Antp protein levels by immunostaining. How does Antp specify flight AMPs? Although it was clearly demonstrated that the specification of embryonic abdominal AMPs depends on the Notch/Numb pathway, no studies addressed the specification of thoracic AMPs (*53*–*55*). Whether the thoracic AMP specification depends on *Notch* and how *Antp* intervenes in this process remains unknown.

An interesting point that stems from our findings is that the precise modulation of *Hox* gene expression in muscle precursors over time is crucial for the proper muscle fiber fate. It has been reported that ectopic *Antp* expression in wing myoblasts causes the absence of adult flight muscles, interpreted as *Hox* expression in flight muscle precursors being disruptive for flight myogenesis (*10*). We offer an alternative explanation by showing that, while, on the one hand, Antp expression in flight muscle precursors is necessary for their specification, its clearance at the beginning of metamorphosis is also necessary for fibrillar versus tubular fate acquisition. Furthermore, while *vg* was shown to activate *salm* expression in postfusion myoblasts, the overexpression of *vg* and its cofactor *scalloped* (*sd*) is not sufficient to induce ectopic Salm expression in wing disc myoblasts (*47*). We believe that this is caused by the concomitant repression of *salm* by Antp in these cells, although we did not address whether Antp binds directly to the *salm* promoter in myoblasts due to their low number and a lack of chromatin immunoprecipitation–grade appropriate antibodies.

At the molecular level, we believe that the fate choice between thoracic tubular and fibrillary muscles is linked with the differential usage of the two *Antp* promoters in distinct thoracic segments. While AMPs in the T2 segment express *Antp* from the P2 promoter, AMPs of the T3 segment express *Antp* from the P1 promoter as they were not affected by the *Antp^P2-null^* mutant condition (see Fig. 2). As extensively suggested before (*11*), we believe that this differential promoter usage allows for distinct regulatory inputs between segments, allowing for the Antp T2-specific mesodermal clearance in the early pupa, necessary for the fibrillary muscle development.

In conclusion, this work corrects the long-accepted postulate stipulating that the establishment of thoracic muscles would be independent of *Hox* genes and instead establishes a broad master regulatory role, orchestrating several key steps of adult muscle development: progenitor specification, myoblast fusion, muscle splitting, early sarcomerogenesis, and fibrillar versus tubular fating.

## MATERIALS AND METHODS

### Fly stocks

Flies were raised under standard conditions at 25°C, unless otherwise stated, in a 12-hour light/12-hour dark cycle. For the manipulation of Antp expression, the following lines were used: P2-mutant allele *Antp^s1^* (BL290), *Antp null-allele Antp^ns-rvc3^* (BL1829), Antp RNAi #1 (BL27675), Antp RNAi #2 (109547/KK, VDRC), Antp RNAi #3 (BL64926), and UAS-Antp (*56*). For Antp visualization, *Antp P1-*Gal4 (BL26817), *Antp P2-*Gal4 (this study, described below), *Antp*-Gal4 (*57*), and *Antp::GFP* (gift from S. Merabet) lines were used. With the exception of RNA-seq, all experiments were performed with all three RNAi lines.

Muscle-specific Gal4 lines used are *Mef2-*Gal4 (BL27390), *1151-*Gal4 (*45*), *Act88F-*Gal4 (*45*), and *Him-*Gal4 (*29*). UAS lines used are: *UAS-mCherry nls* (BL38424), *UAS-GFP nls* (BL4776), *UAS-Mlc-HA* (described below), and *UAS-GFP::Gma* where the actin-binding domain of Moesin is fused to GFP (*58*). Reporters used are the *weeP26* transgene (*Myosin-GFP*) containing an insertion of GFP in the *mhc* gene (*59*) and *salm::GFP* (*60*). Wild-type (WT) flies are the Oregon-R strain.

For myoblast counting and RNA-seq analysis, larvae were staged to 0 hours APF, where the number of myoblast is the highest (*16*). For optimal penetrance, all experiments with flies expressing *Mef2-GAL4* x *Antp RNAi* #1 and #2 were performed at 29°C. All other crosses were performed at 25°C.

### Generation of the UAS-Mlc2-HA line

The UAS-Mlc2 line was constructed by cloning the Mlc2 coding sequence obtained from the FlyBase database (*61*) in the pUAST plasmid containing the hemagglutinin (HA) tag using standard cloning procedures. Briefly, the Mlc2 coding sequence was recovered from the clone RE01159 [Drosophila Genomics Resource Center (DGRC) stock no. 1074315; https://dgrc.bio.indiana.edu//stock/1074315; RRID:DGRC_1074315] by polymerase chain reaction (PCR) amplification, using the following primers: forward 5′-AAAAGAATTCATGGCCGATGAGAAGAAG-3′ and reverse 5′-TTTTCTCGAGGGCGGCCTCCTCCTCCTC-3′, adding Eco RI and Xho I restriction sites, respectively. The purified PCR product was digested using Eco RI (FD0278, Thermo Fisher Scientific) and Xho I (FD0698, Thermo Fisher Scientific) restriction enzymes using the FastDigest protocol. The digested product was cloned into an Eco RI–Xho I-digested pUASt vector using T4 ligase (M180A, Promega) with a vector-to-insert ratio of 1:3, overnight (ON) at 4°C. The ligated product was transformed into competent bacteria by heat shock and verified by sequencing.

### Generation of the Antp P2-Gal4 line

For the construction of the *Antp P2-*Gal4 line, a minimal sequence of ~6 kb [5738-bp upstream of the transcription start site (TSS) plus 250 bp of exon 3], shown to drive expression from the Antp P2 promoter [*5.6d9*, described in (*22*)], was cloned in the pPTGAL vector (DGRC stock no. 1225; https://dgrc.bio.indiana.edu//stock/1225; RRID:DGRC_1225) using the In-Fusion cloning kit (639649, Takara). Briefly, the Antp P2 regulatory sequences were recovered from a BAC clone (CH321-43D16, BACPAC Resources Center) by PCR. The sequence upstream of TSS plus 250 bp of exon 3 was amplified in two fragments using the following primers: 5′-GCTGAACAAGCTAAACAATCTGCAGGTACCATTCATTTTTCTTGACTATTTTGG-3′ 5′-CACGGGAAATGAAACTGAAAAGGGAAAGGAAAC-3′ and 5′-CAGTTTCATTTCCCGTGCGCCCAAAGTTTCC-3′ 5′-TTGGGCTGCAGCCTCGTCCTGAGCAGGCAGCGAA-3′. These fragments were inserted in the Pst I site upstream the translation start site of Gal4.

### Immunofluorescence

#### Embryos

Embryos were collected on agar plates, dechorionated using 50% bleach for 3 min, and fixed in a 4% formaldehyde solution containing heptane. Embryos were dehydrated in a methanol wash followed by three ethanol washes, rehydrated in 50% ethanol/50% PBT [0.1% Tween and phosphate-buffered saline (PBS)], washed for 15 min in PBT, saturated for 1 hour in 10% bovine serum albumin (BSA) in PBT, and incubated with primary antibodies in PBT ON at 4°C. Embryos were washed three times in PBT, incubated with secondary antibodies for 1.5 hours in PBT containing Hoechst (1:1000, H3570, Life Technologies) at room temperature (RT) and rewashed three times in PBT.

#### Imaginal discs

Larvae were dissected using forceps (Fine Science Tools, Dumont #5) in PBS and fixed for 20 min in 4% formaldehyde in PBS, rinsed, and washed three times in PBT (0.2% Triton X-100 and PBS) for 10 min each. Samples were blocked in 2% BSA in PBT (blocking solution) for at least 1 hour and incubated with primary antibodies in the blocking solution ON at 4°C. After three washes in PBT, samples were incubated with secondary antibodies in the blocking solution containing Hoechst (1:1000, H3570, Life Technologies) for 1.5 hours followed by washing six times in PBT for 10 min each.

#### Indirect flight muscles

Pupal flight muscles were dissected as described in (*32*). Briefly, pupae were collected at 0 hours APF and stuck to a slide using double-sided tape for staging. Pupae were removed from the pupal case using forceps, poked at the thorax/abdomen junction, and fixed in 4% formaldehyde in PBS for 20 min. Pupae were then pinned at the abdomen to a dissecting plate containing PBS and cut open using fine scissors (no. 15003-08, Fine Science Tools), isolating flight muscles attached to the cuticle. Quarter thoraces were then washed in PBT (0.2% Triton X-100 and PBS) for 10 min and saturated in 2% BSA for at least 1 hour. Samples were incubated with primary antibodies in the blocking solution ON at 4°C and washed three times in PBT for 10 min each and with secondary antibodies in PBT containing Hoechst (1:1000, H3570, Life Technologies) for 1.5 hours at RT, followed by six washes in PBT. Pupae younger than 48 hours APF were mounted as wing discs; for older pupae, one or more layers of double-sided tape were used as spacers to prevent tissue deformation.

For adult flight muscles, females were quickly rinsed in ethanol, and wings, head, and abdomen were removed in PBS. For the visualization of DLMs, thoraces were split in half using forceps; for dissection of DVMs, DLMs were removed using forceps and the excess cuticle was removed using fine scissors. For DFM dissection, DLMs, DVMs, and the jump muscle [tergal depressor of the trochanter muscle (TDT)] were removed using forceps and the excess cuticle was removed using fine scissors. Samples were fixed in 4% formaldehyde in PBS for 20 min, rinsed, and washed three times in PBT (0.5% Triton X-100 and PBS) for 10 min each and blocked in 4% normal goat serum (S26-M, Sigma-Aldrich) in PBT for at least 1 hour. Thoraces were incubated ON with primary antibodies in PBT at 4°C. Samples were rinsed and washed three times in PBT for 15 min each, incubated with secondary antibodies in PBT including phalloidin (1:500, phalloidin–Atto 647N, Sigma-Aldrich) and Hoechst (1:1000, H3570, Life Technologies) at RT for 2 hours, and washed again six times in PBT for 10 min each. All steps were performed in a 24-well plate under agitation.

For all tissues, samples were mounted in a Vectashield medium (Vector Laboratories) and imaged using a confocal microscope (Zeiss LSM 880). Primary antibodies used were anti-Antp (1:100 for embryos and discs and 1:20 for pupae, 8c11, Developmental Studies Hybridoma Bank (DSHB), anti-Antp (1:50, 4c3, DSHB), anti-GFP (1:500, GFP-1010, Aves Labs), and rabbit anti-Twi (1:200, gift from E. Furlong). Secondary antibodies were coupled to Alex Fluor 488, 568, or 647 (1:500, Life Technologies).

### Live pupal IFM imaging

Live pupal flight muscle imaging was performed as described in (*32*) using a spinning disc microscope (Spinning ROPER, Zeiss). Briefly, samples were taped on a slide using double-sided tape with three coverslips placed on each side to prevent tissue deformation. Pupae were covered in 50% glycerol and imaged ON at 29°C every 10 min. Movies are available in the Supplementary Materials.

### Transmission electron microscopy

Pupal and adult flight muscle samples were processed as described in (*62*) and imaged on a transmission electron microscope FEI Tecnai G2 at 200 kV or FEI Tecnai T12 at 120 kV.

### Flight assay

To assess flight capacity, 3- to 4-day-old 15 males per genotype were thrown into a Plexiglas cylinder (with the height of 1 m and a diameter of 8.4 cm), and the landing position in the cylinder (top, middle, and bottom) was scored (*32*). Flies landing at the top are considered as able to fly, in the middle as weak flyers, and to the bottom as flightless. At least 170 flies were tested per genotype, and three independent biological replicates were performed.

### Sample preparation for RNA-seq and high-throughput analysis

For FACS-sorted wing disc–associated myoblasts and pupal flight muscles, samples were prepared and analyzed as described previously (*62*). DEGs were called using DESeq2 using a false discovery rate (adjusted *P* value in DESeq2) threshold of 0.001. The lists of DEGs for all RNA-seq datasets are available in data S1 to S3. GO analyses were performed using the RStudio software (http://www.rstudio.com/) on DEGs, using the enrichGO function from clusterProfiler. For all samples, RNA quality was verified via Bioanalyzer (Agilent).

### Quantifications and statistical analysis

All image processing and quantifications were performed using the Fiji software (*63*). The intensity of Antp staining in myoblasts was quantified as a ratio of the Antp intensity in the Twi-positive myoblast area and in the disc epithelium (the background fluorescence was subtracted). The number of wing disc–associated myoblasts was quantified using a three-dimensional nuclei count tool (*64*) as described in (*62*). The number of pupal myoblasts was quantified manually in a defined volume of 14,034 μm^3^. The length of pupal fibers was quantified measuring the distance between two ends of the longest muscle fiber for each sample. Muscle parameters, sarcomere length, and myofibril width were estimated using the MyofibrilJ plugin for ImageJ (https://imagej.net/MyofibrilJ) (*29*). In adult flight muscles, the intensity of Salm staining was measured with region of interest applied on nuclei. Statistical tests and charts were performed using the RStudio software. In Fig. 3E, an analysis of variance (ANOVA) test for multiple comparison was used followed by Tukey post hoc test (*P* value_y_RNAi vs #1_ = 0.42, *P* value_y_RNAi vs #2_ = 0.99, and *P* value_y_RNAi vs #3_ = 0.79). In Fig. 3F, an unpaired Student’s test was applied (*P* value = 0.75). In fig. S4D, after applying a Shapiro-Wilk test for normality, a nonparametric, Mann-Whitney test was applied [*P* values (length) = 0.007 and 0.4 × 10^−8^ and *P* values (width) = 0.03 and 0.9 × 10^−8^]. In fig. S4F, the same test as in fig. S4D was applied [*P* value (length) = 0.02 and *P* value (width) = 0.003]. All experiments were performed in one technical replicate and three biological replicates except for the RNA-seq analyses that were performed in three technical replicates and one biological replicate.
