# Supplementary material for: The Hox protein Antennapedia orchestrates Drosophila adult flight muscle development
Source: Sci Adv. 2024 Nov 27;10(48):eadr2261. doi: 10.1126/sciadv.adr2261 (PMC11601212; doi:10.1126/sciadv.adr2261)
Supplement: Supplementary file 1 — Figs. S1 to S9 Legends for movies S1 to S3 Legends for data S1 to S3 [file sciadv.adr2261_sm.pdf]

Science Advances
AAAS

Supplementary Materials for

The Hox protein Antennapedia orchestrates *Drosophila* adult flight muscle development

Gabriela Poliacikova *et al.*

Corresponding author: Andrew J. Saurin, andrew.saurin@univ-amu.fr

Sci. Adv. **10**, eadr2261 (2024)
DOI: 10.1126/sciadv.adr2261

The PDF file includes:

Figs. S1 to S9
Legends for movies S1 to S3
Legends for data S1 to S3

Other Supplementary Material for this manuscript includes the following:

Movies S1 to S3
Data S1 to S3

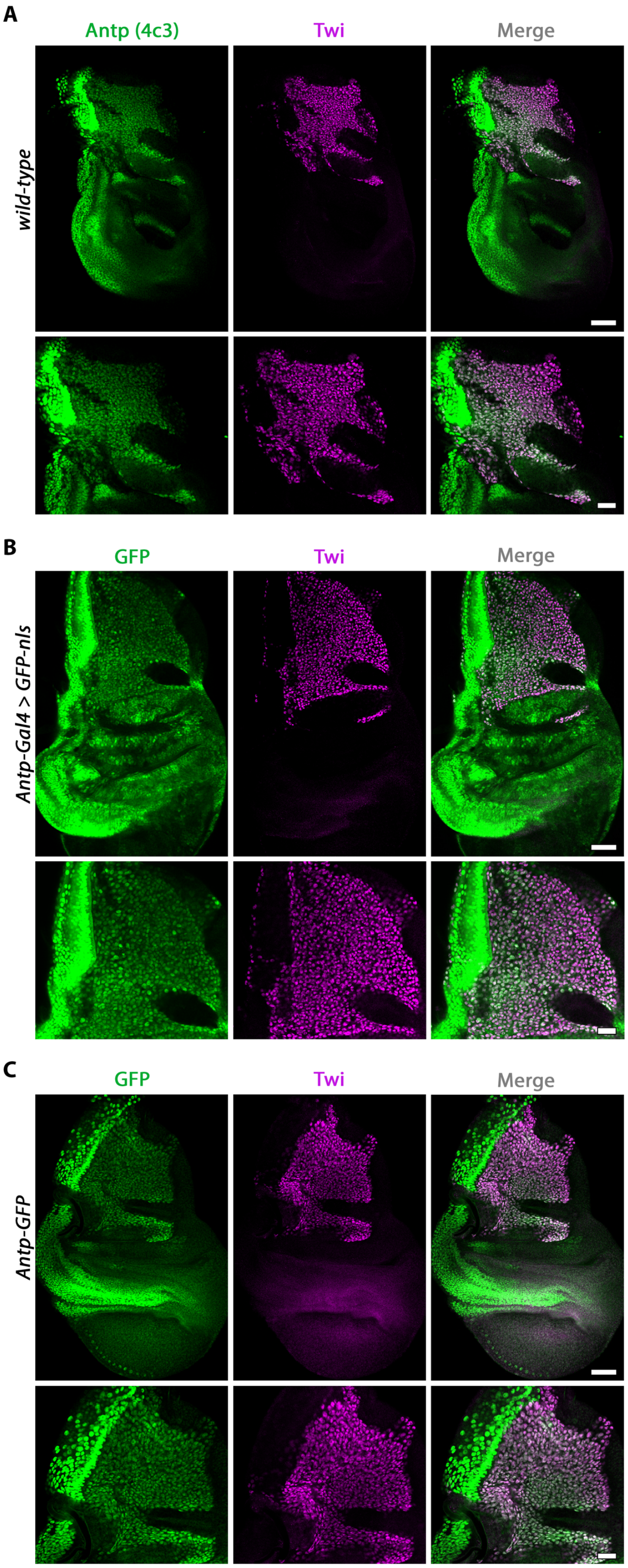
A
Antp (4c3)
Twi
Merge
wild-type
B
GFP
Twi
Merge
Antp-Gal4 > GFP-nls
C
GFP
Twi
Merge
Antp-GFP

Fig. S1. Antp is expressed in larval flight muscle myoblasts.

(A) Confocal sections of wild-type wing discs at the L3 wandering stage, stained with antibodies against Antp (4c3) and Twi. **(B)** Confocal sections of 0 h APF wing discs expressing *UAS-GFP nls* driven by *Antp*-Gal4, stained with antibodies against GFP and Twi. **(C)** Confocal sections of 0 h APF wing discs expressing *Antp-GFP*, stained with antibodies against GFP and Twi. The scale bar is 50 μm for the top and 20 μm for the bottom views in all panels.

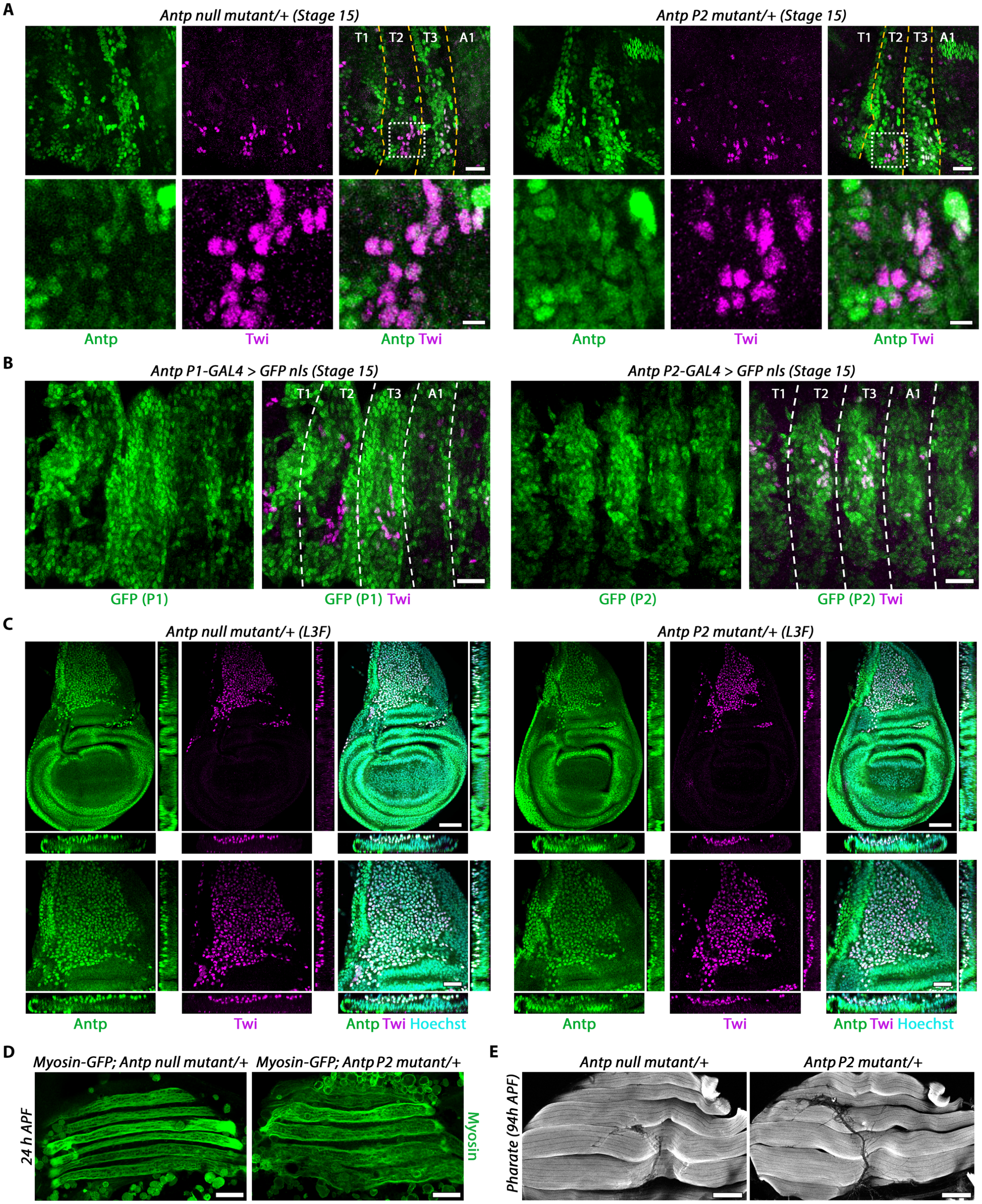

A
Antp null mutant/+ (Stage 15)
Antp P2 mutant/+ (Stage 15)
T1
T2
T3
A1
Antp
Twi
Antp Twi
B
Antp P1-GAL4 > GFP nls (Stage 15)
Antp P2-GAL4 > GFP nls (Stage 15)
GFP (P1)
GFP (P1) Twi
GFP (P2)
GFP (P2) Twi
C
Antp null mutant/+ (L3F)
Antp P2 mutant/+ (L3F)
Antp
Twi
Antp Twi Hoechst
D
Myosin-GFP; Antp null mutant/+
Myosin-GFP; Antp P2 mutant/+
24 h APF
Myosin
E
Antp null mutant/+
Antp P2 mutant/+
Pharate (94h APF)

Fig. S2. Heterozygous *Antp* mutations do not perturb flight muscle development.

(A) Confocal sections of stage 15 embryos labelled with antibodies against Antp and Twi. Genotypes are heterozygous $Antp^{ns\text{-}rvc1}$ (null mutation) (Left) and heterozygous $Antp^{s1}$ (P2 mutation) (Right). The scale bar is 20 µm for top views and 5µm for zoomed, bottom views on T2 AMPs, depicted by the white dashed square. **(B)** Confocal sections of stage 15 embryos expressing *UAS GFP-nls* driven by the *Antp P1*-Gal4 (Left) and *Antp P2*-Gal4 (Right), stained with anti-GFP and anti-Twi antibodies. The scale bar is 20µm. In (A) and (B) T1, T2, T3 depict the three thoracic segments and A1 the first abdominal segment. **(C)** Confocal sections of wing discs at the L3 feeding stage labelled with antibodies against Antp and Twi. Genotypes are identical to (A). The scale bar is 50 µm for large and 25 µm for zoomed views. **(D)** Confocal projections of 24 h APF pupal IFMs labelled with GFP to visualise myosin. Genotypes are identical to (A) with *weeP26* transgene (Myosin-GFP) added. The scale bar is 50µm. **(E)** Confocal sections of pupal IFM at the pharate stage, labelled with phalloidin. Genotypes are identical to (A). The scale bar is 100 µm.

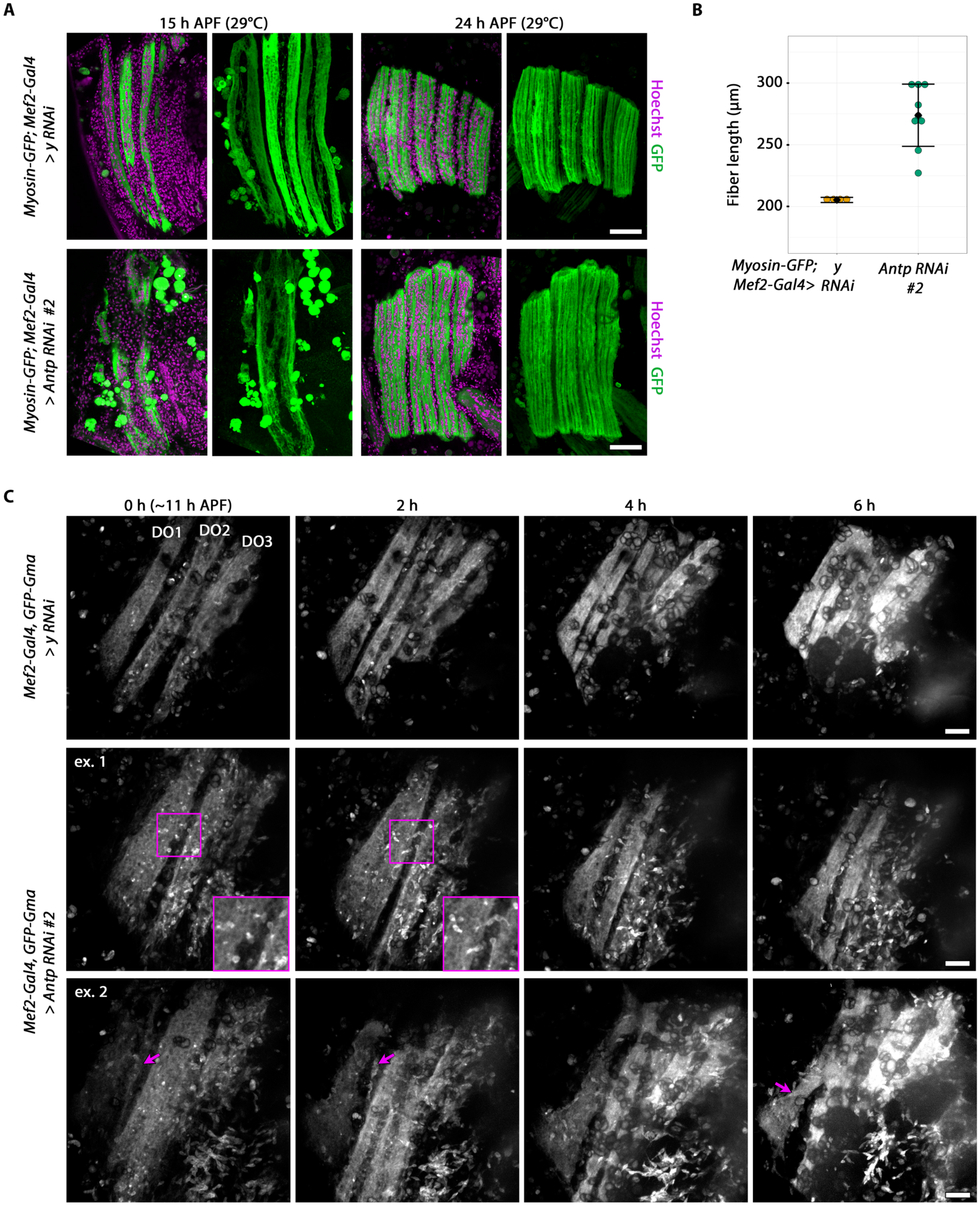
A
15 h APF (29°C)
24 h APF (29°C)
Myosin-GFP; Mef2-Gal4 > y RNAi
Myosin-GFP; Mef2-Gal4 > Antp RNAi #2
Hoechst GFP
Hoechst GFP
B
Fiber length (µm)
300
250
200
Myosin-GFP; Mef2-Gal4>
y RNAi
Antp RNAi #2
C
0 h (~11 h APF)
2 h
4 h
6 h
DO1
DO2
DO3
Mef2-Gal4, GFP-Gma > y RNAi
Mef2-Gal4, GFP-Gma > Antp RNAi #2
ex. 1
ex. 2

Fig. S3. ***Antp*** **KD perturbs pupal flight muscle development.**

(A) Confocal sections of 15 h and 24 h APF flight muscles expressing *weeP26; Mef2*-GAL4 driving *UAS-y RNAi* as a control (top) or *UAS-Antp RNAi #2* (bottom). Flight muscles are stained with an anti-GFP antibody to visualize myosin and Hoechst. Scale bar is 50 µm. **(B)** Quantification of flight muscle fibre length at 24 h APF, genotypes are identical to (A). Mean and standard deviation are shown, each dot represents a value from one animal ($n_{WT} = 4$, $n_{\#2} = 9$ animals). **(C)** Spinning disc, timecourse sections of developing pupal flight muscles. Genotypes are *UAS-y RNAi* (control condition) and *UAS-Antp RNAi #2* driven by the *Mef2*-GAL4 driver, with the *GPF-Gma* transgene added, labelling actin. Two examples of the RNAi condition are shown (Middle and Bottom). Zoomed views show myoblasts delayed in their fusion (ex. 1) and arrows point to detaching muscle fibres (ex. 2). DO – dorsal oblique muscles

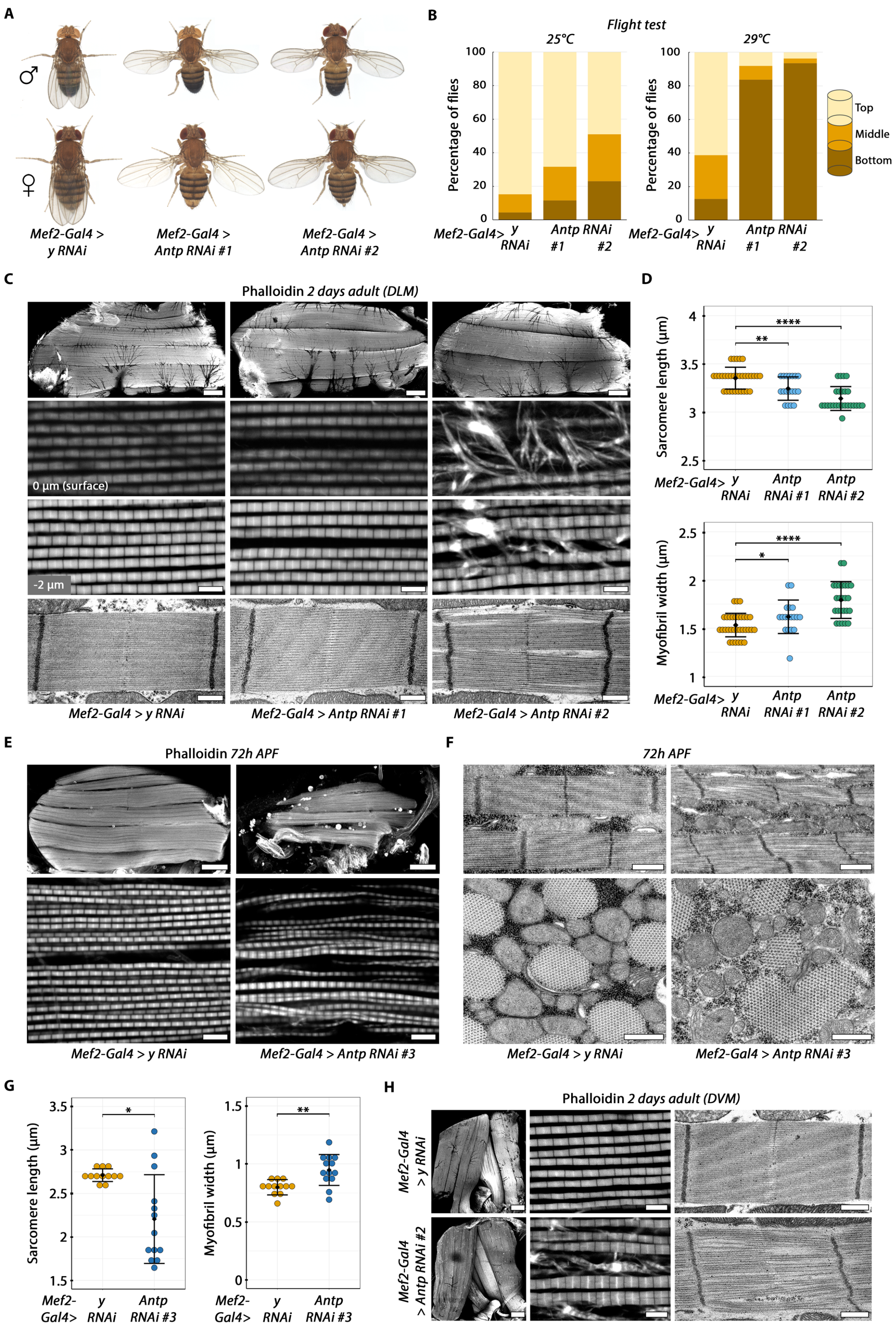

A
♂
♀
Mef2-Gal4 > y RNAi
Mef2-Gal4 > Antp RNAi #1
Mef2-Gal4 > Antp RNAi #2
B
Flight test
25°C
29°C
Percentage of flies
Top
Middle
Bottom
Mef2-Gal4> y RNAi
Antp RNAi #1 #2
C
Phalloidin 2 days adult (DLM)
0 µm (surface)
-2 µm
Mef2-Gal4 > y RNAi
Mef2-Gal4 > Antp RNAi #1
Mef2-Gal4 > Antp RNAi #2
D
Sarcomere length (µm)
Myofibril width (µm)
Mef2-Gal4> y RNAi
Antp RNAi #1
Antp RNAi #2
E
Phalloidin 72h APF
Mef2-Gal4 > y RNAi
Mef2-Gal4 > Antp RNAi #3
F
72h APF
Mef2-Gal4 > y RNAi
Mef2-Gal4 > Antp RNAi #3
G
Sarcomere length (µm)
Myofibril width (µm)
Mef2-Gal4> y RNAi
Antp RNAi #3
H
Phalloidin 2 days adult (DVM)
Mef2-Gal4 > y RNAi
Mef2-Gal4 > Antp RNAi #2

Fig. S4. *Antp* KD perturbs adult flight muscle myofibrillogenesis.
(A) Images of adult flies expressing *UAS-y RNAi* (as a control) or *UAS-Antp RNAi* (# depicts two different RNAi lines) driven by the *Mef2*-Gal4 driver, at 29°C. **(B)** Flight ability was scored as a percentage of male flies landing on each section of the column (top, middle or bottom section). The test was perfomed on flies kept at 25°C (n_{y_RNAi}=176, $n_{Antp\_RNAi\_\#1}$ =261 and $n_{Antp\_RNAi\_\#2}$ =216 flies) and at 29°C (n_{y_RNAi}=202, $n_{Antp\_RNAi\_\#1}$ =223 and $n_{Antp\_RNAi\_\#2}$ =202 flies). **(C)** Confocal sections of adult female DLM stained with phalloidin (Top), with zoomed views on sarcomeres and their surface (Middle). Scale bars are 100 µm for large and 5 µm for zoomed views. TEM micrographs of adult DLM sarcomeres are shown at the bottom. The scale bar is 500 nm. Genotypes are identical to (A). **(D)** Quantification of muscle parameters, sarcomere length (Top) and myofibril width (Bottom). Each point corresponds to a value obtained from one animal, mean and standard error are indicated (n_{y_RNAi}=32, $n_{Antp\_RNAi\_\#1}$ =18 and $n_{Antp\_RNAi\_\#2}$ =25 flies). Genotypes are identical to (A). **(E)** Confocal sections of pupal DLM at 72 h APF stained with phalloidin (Top), with zoomed view on sarcomeres (Bottom), at 25°C. Scale bars are 100 µm for large and 5 µm for zoomed views. Genotypes are *UAS-y RNAi* (as a control) and *UAS-Antp RNAi #3* driven by the *Mef2*-Gal4 driver. **(F)** TEM micrographs of pupal sarcomeres at 72h APF, cut in longitudinal (Top) and cross-sections (Bottom). The scale bar is 500 nm for all images. Genotypes are identical to (E). **(G)** As in (D) with (n_{y_RNAi}=12 and $n_{Antp\_RNAi\_\#3}$ =13 flies). Genotypes are identical to (E). **(H)** Confocal sections of adult female DVM stained with phalloidin (Left), with zoomed views on sarcomeres (Middle). Scale bars are 100 µm for large and 5 µm for zoomed views. TEM micrographs of adult DVM sarcomeres are shown on the right. The scale bar is 500 nm. Genotypes are *UAS-y RNAi* (as a control) and *UAS-Antp RNAi #2* driven by the *Mef2*-Gal4 driver.

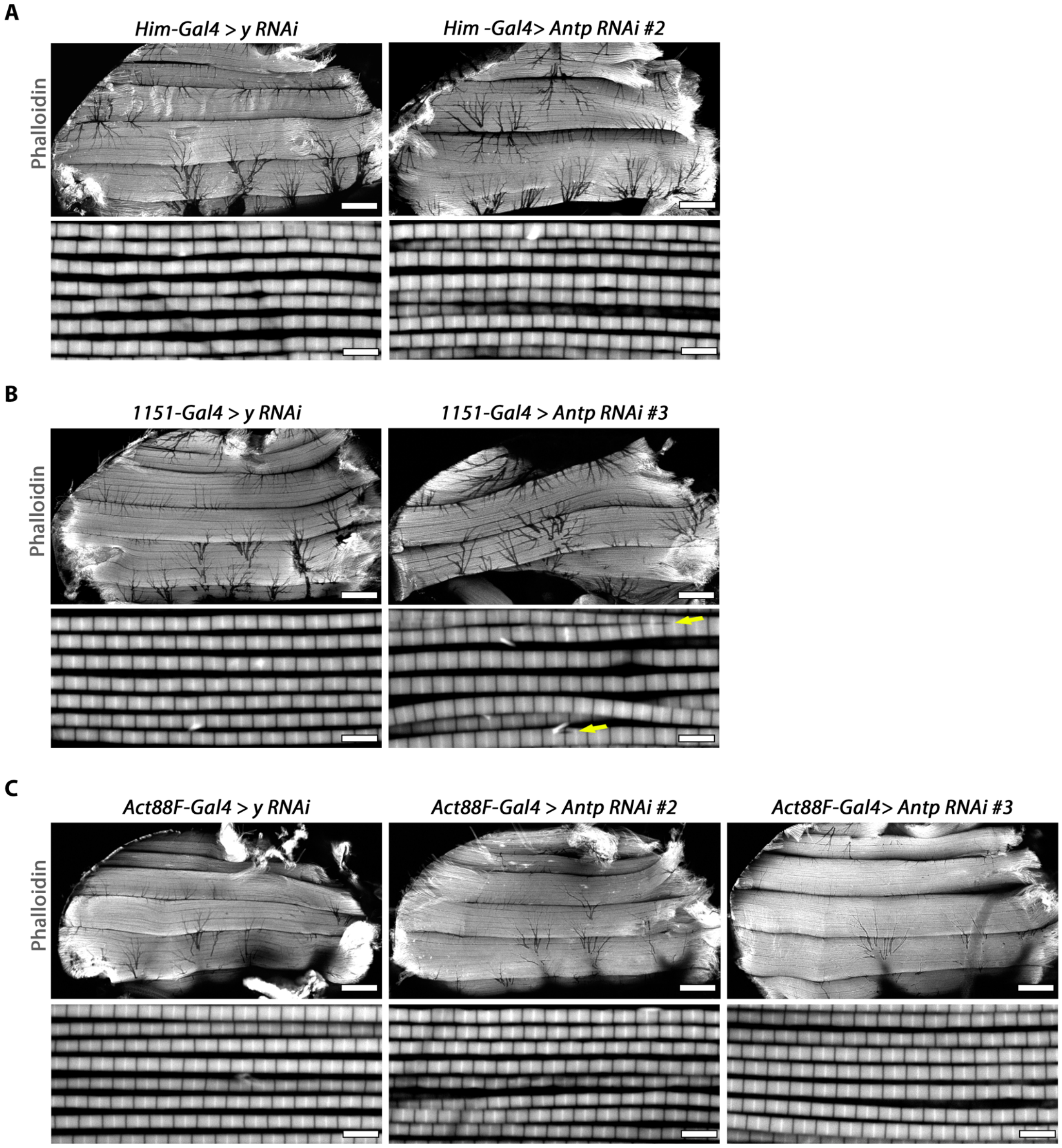
A
Him-Gal4 > y RNAi
Him -Gal4> Antp RNAi #2
Phalloidin
B
1151-Gal4 > y RNAi
1151-Gal4 > Antp RNAi #3
Phalloidin
C
Act88F-Gal4 > y RNAi
Act88F-Gal4 > Antp RNAi #2
Act88F-Gal4> Antp RNAi #3
Phalloidin

Fig. S5. *Antp* early pupal, but not late pupal downregulation perturbs adult flight myogenesis.

(A, B, C) Confocal sections of adult female DLM stained with phalloidin (Top), with zoomed views on sarcomeres (Bottom). Genotypes are (A) *UAS-y RNAi* (as a control), *UAS-Antp RNAi #2* driven by the *Him*-Gal4 driver showing defects in the number of DLM fibers upon Antp RNAi. (B) *UAS-y RNAi* (as a control), *UAS-Antp RNAi #3* driven by the *1151*-Gal4 driver, maintained at 29°C, showing splitting of myofibrils (arrow). (C) *UAS-y RNAi*, *UAS-Antp RNAi #2, UAS-Antp RNAi #3* driven by the *Act88F*-Gal4 driver results in no visible defects to DLM development. Scale bars are 100 µm for large and 5 µm for zoomed views.

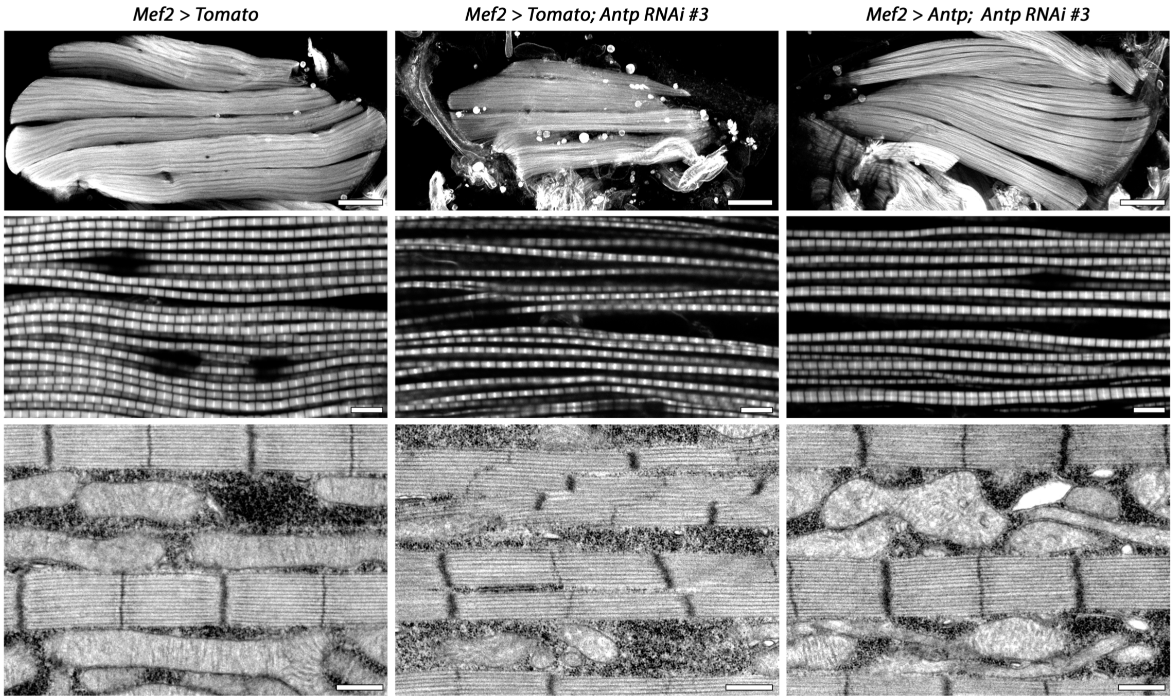

Mef2 > Tomato
Mef2 > Tomato; Antp RNAi #3
Mef2 > Antp; Antp RNAi #3

Fig. S6. Defaults in pupal flight muscle development in the *Antp* KD can be rescued with *Antp* overexpression.

Confocal sections of female pupal DLM at 72h APF stained with phalloidin (Top), with zoomed views on sarcomeres (Middle). Scale bars are 100 µm for large and 5 µm for zoomed views. TEM micrographs of 72h pupal sarcomeres are shown at the bottom. The scale bar is 500 nm. Genotypes are *UAS-Tomato* (Left), *UAS-Tomato; UAS-Antp RNAi #3* (Middle) and *UAS-Antp; UAS-Antp RNAi #3* (Right), driven by the *Mef2*-Gal4 driver.

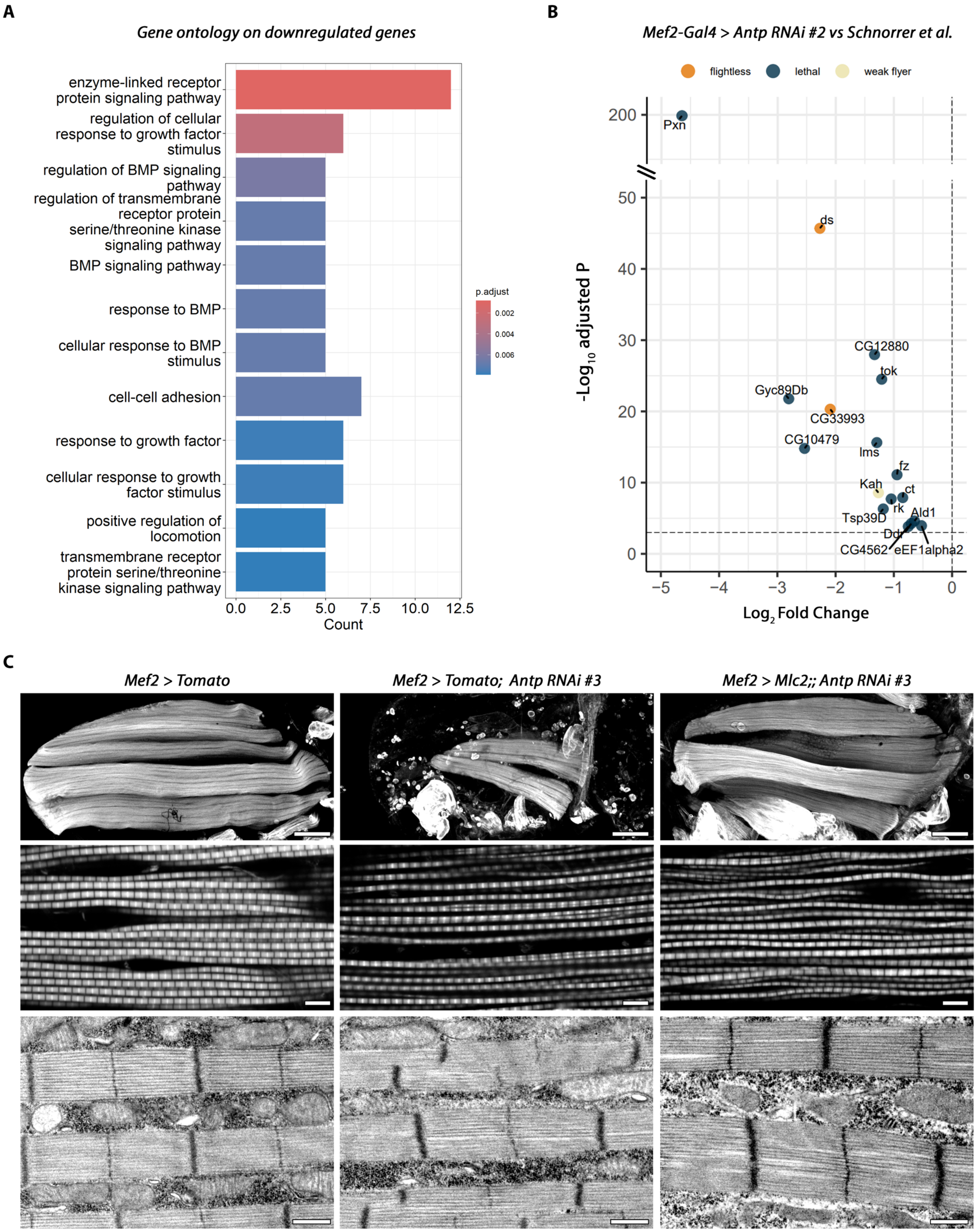
A
Gene ontology on downregulated genes
enzyme-linked receptor protein signaling pathway
regulation of cellular response to growth factor stimulus
regulation of BMP signaling pathway
regulation of transmembrane receptor protein serine/threonine kinase signaling pathway
BMP signaling pathway
response to BMP
cellular response to BMP stimulus
cell-cell adhesion
response to growth factor
cellular response to growth factor stimulus
positive regulation of locomotion
transmembrane receptor protein serine/threonine kinase signaling pathway
0.0
2.5
5.0
7.5
10.0
12.5
Count
p.adjust
0.002
0.004
0.006
B
Mef2-Gal4 > Antp RNAi #2 vs Schnorrer et al.
flightless
lethal
weak flyer
200
Pxn
50
40
30
20
10
0
ds
CG12880
tok
Gyc89Db
CG33993
CG10479
lms
fz
Kah
ct
rk
Tsp39D
Ald1
Dd
CG4562
eEF1alpha2
−5
−4
−3
−2
−1
0
-Log10 adjusted P
Log2 Fold Change
C
Mef2 > Tomato
Mef2 > Tomato; Antp RNAi #3
Mef2 > Mlc2;; Antp RNAi #3

Fig. S7. Defaults in pupal flight muscle development in the *Antp* KD can be rescued with *Mlc2* overexpression.

(A) Gene ontology analysis using significantly downregulated genes upon *Antp* KD in larval myoblasts. **(B)** Volcano plot showing significantly downregulated genes upon *Antp* KD having a phenotype in the RNAi screen from *Schnorrer et al. 2010* (*40*). **(C)** Confocal sections of female pupal DLM at 72h APF stained with phalloidin (Top), with zoomed views on sarcomeres (Middle). Scale bars are 100 µm for large and 5 µm for zoomed views. TEM micrographs of 72h pupal sarcomeres are shown at the Bottom. The scale bar is 500 nm. Genotypes are *UAS-Tomato* (Left), *UAS-Tomato; UAS- Antp RNAi #3* (Middle) and *UAS-Mlc2;; UAS- Antp RNAi #3* (Right) driven by the *Mef2-Gal4* driver.

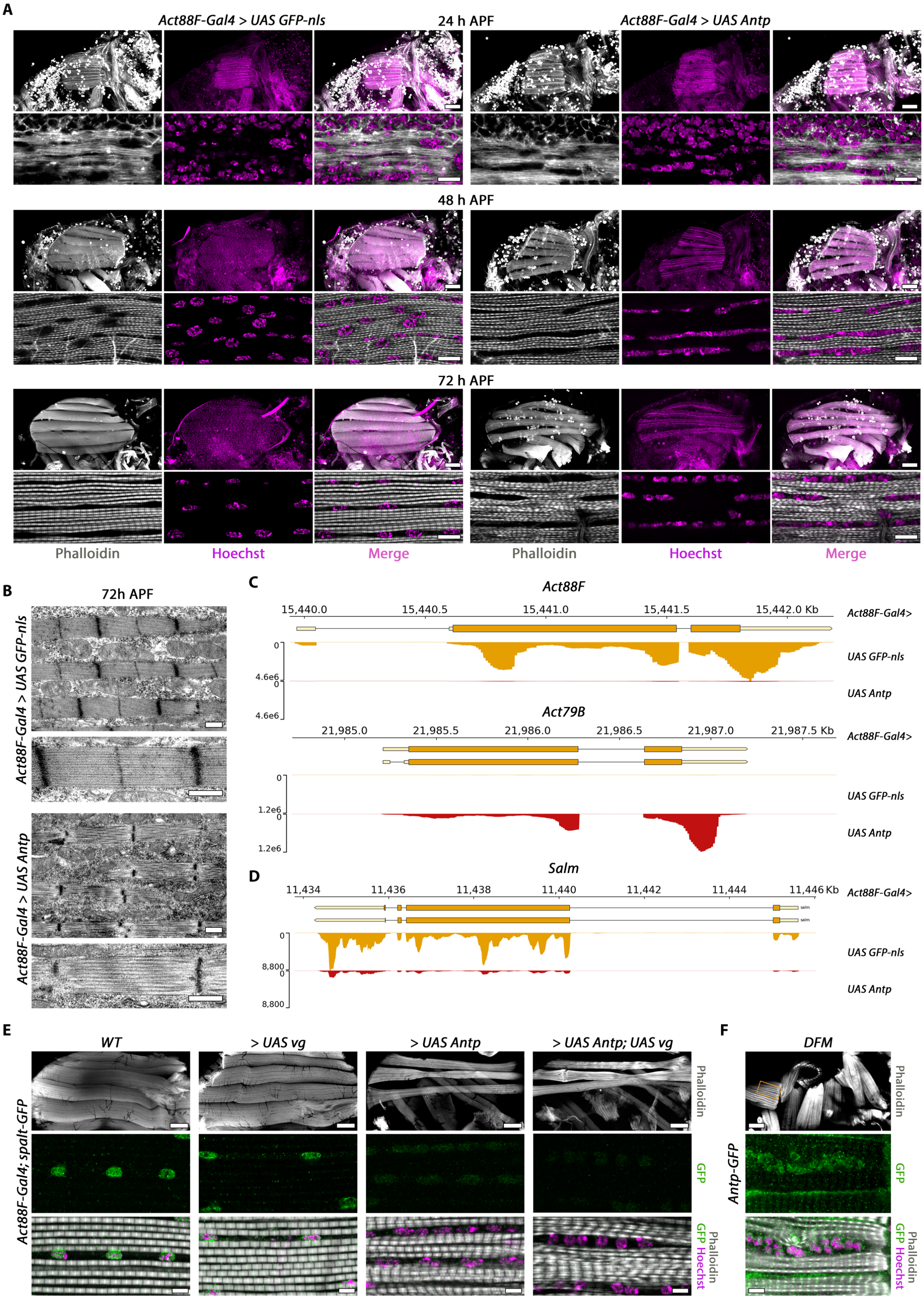
A
Act88F-Gal4 > UAS GFP-nls
24 h APF
Act88F-Gal4 > UAS Antp
48 h APF
72 h APF
Phalloidin
Hoechst
Merge
Phalloidin
Hoechst
Merge
B
72h APF
Act88F-Gal4 > UAS GFP-nls
Act88F-Gal4 > UAS Antp
C
Act88F
15,440.0
15,440.5
15,441.0
15,441.5
15,442.0 Kb
Act88F-Gal4>
0
4.6e6
0
4.6e6
UAS GFP-nls
UAS Antp
Act79B
21,985.0
21,985.5
21,986.0
21,986.5
21,987.0
21,987.5 Kb
Act88F-Gal4>
0
1.2e6
0
1.2e6
UAS GFP-nls
UAS Antp
D
Salm
11,434
11,436
11,438
11,440
11,442
11,444
11,446 Kb
Act88F-Gal4>
0
8,800
0
8,800
UAS GFP-nls
UAS Antp
E
WT
> UAS vg
> UAS Antp
> UAS Antp; UAS vg
Act88F-Gal4; spalt-GFP
Phalloidin
GFP
GFP Hoechst Phalloidin
F
DFM
Antp-GFP
Phalloidin
GFP
GFP Hoechst Phalloidin

Fig. S8. *Antp* overexpression prevents fibrillar fibre fate.

(A) Confocal sections of pupal DLM at 24 h (Top), 48 h (Middle) and 72 h APF (Bottom), stained with phalloidin and Hoechst. For each timepoint zoomed views of sarcomeres are shown. Scale bars are 100 µm for large and 10 µm for zoomed views. Genotypes are *UAS-GFP-nls* (Left panel) and *UAS-Antp* (Right panel) driven by the *Act88F*-Gal4 driver. **(B)** TEM micrographs of 72h APF DLM, cut in longitudinal section, with a zoomed view on a sarcomere. The scale bar is 500 nm for all micrographs. Genotypes are identical to (A). **(C)** Genome browser images and normalised RNA-seq reads of *Act88F*-Gal4-driven *UAS-GFP* (gold tracks) or *UAS-Antp* (red tracks) for the fibrillar-specific *Act88F* gene (top) and tubular-specific *Act79B* gene (bottom) showing repression of *Act88F* and expression of *Act79B* upon Antp expression. **(D)** Genome browser image and normalised RNA-seq reads of *Act88F*-Gal4-driven *UAS-GFP* (gold tracks) or *UAS-Antp* (red tracks) of the *salm* locus, showing repression of *salm* when Antp is expressed. **(E)** Confocal sections of adult female DLM stained with anti-GFP (to visualise Salm), Phalloidin and Hoechst. Genotypes are wild-type (left), *UAS-Antp* (middle-left), *UAS-vg* (middle-right) and *UAS-Antp*; *UAS-vg* (Right), driven by the *Act88F*-Gal4 driver, combined with the *spalt::GFP* transgene. The scale bar for large IFM views (top) is 100 µm and for zooms on sarcomeres (bottom) 5 µm. **(F)** Confocal sections of female DFM stained with Phalloidin, anti-GFP (to visualise Antp) and Hoechst. The orange rectangle depicts the zoomed area on the DFM muscle 49. The scale bar is 50 µm for the large view (top) and 5 µm for the zoomed view (bottom). The genotype is homozygous *Antp-GFP*.

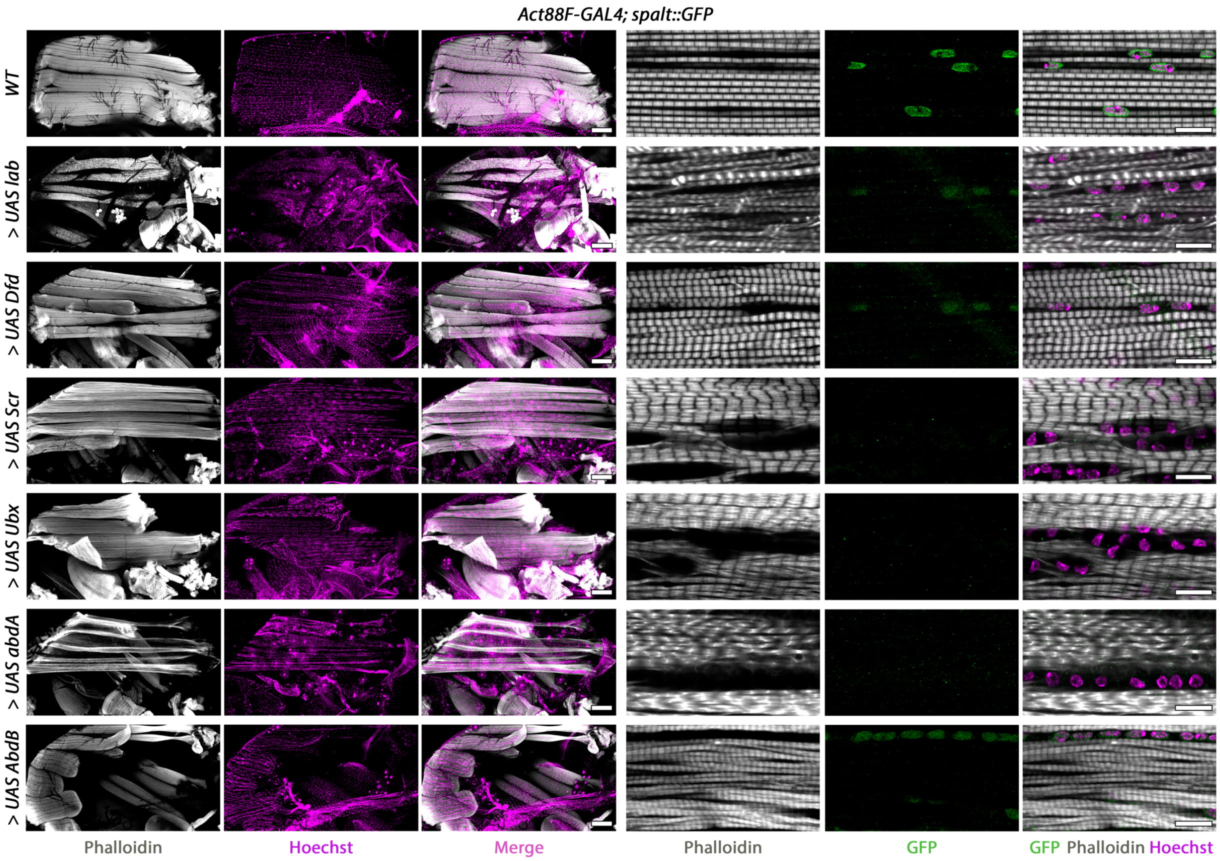
Act88F-GAL4; spalt::GFP
WT
> UAS lab
> UAS Dfd
> UAS Scr
> UAS Ubx
> UAS abdA
> UAS AbdB
Phalloidin
Hoechst
Merge
Phalloidin
GFP
GFP Phalloidin Hoechst

Fig. S9. Fibrillar fate and Spalt repression are shared *Hox* functions.

Confocal sections of adult female DLM stained with anti-GFP (to visualise Salm), Phalloidin and Hoechst. The scale bar is 100 µm for large views and 10 µm for zoomed views on sarcomeres. Genotypes are WT, *UAS-lab*, *UAS-Dfd*, *UAS-Scr*, *UAS-Ubx*, *UAS-abdA* and *UAS-AbdB* driven by the *Act88F*-Gal4 driver.

Movie S1. (separate file)

Spinning disc movie of developing flight muscles at 29°C, expressing *UAS-y RNAi* driven by the *Mef2*-Gal4 *driver,* with the *UAS-GFP-Gma* transgene added to visualise actin.

Movie S2. (separate file)

Spinning disc movie of developing flight muscles at 29°C, expressing *UAS- Antp RNAi #2* driven by the *Mef2*-Gal4 *driver,* with the *UAS-GFP-Gma* transgene added to visualise actin, corresponding to example 1.

Movie S3. (separate file)

Spinning disc movie of developing flight muscles at 29°C, expressing *UAS- Antp RNAi #2* driven by the *Mef2*-Gal4 *driver,* with the *UAS-GFP-Gma* transgene added to visualise actin, corresponding to example 2.

Data S1. (separate file)
Excel file containing differential gene expression analysis of RNA-sequencing following *Antp* RNAi #2 expression in myoblasts, driven by the *Mef2*-Gal4 driver.

Data S2. (separate file)
Excel file containing the comparison of differential gene expression analysis of RNA-sequencing following *Antp* RNAi #2 in myoblasts, driven by the *Mef2*-Gal4 driver, with the screen from *Schnorrer et al. 2010* (*40*).

Data S3. (separate file)
Excel file containing differential gene expression analysis of RNA-sequencing following Antp overexpression driven by the *Act88F*-GAL4 driver in 72 h APF pupa at 25°C.
